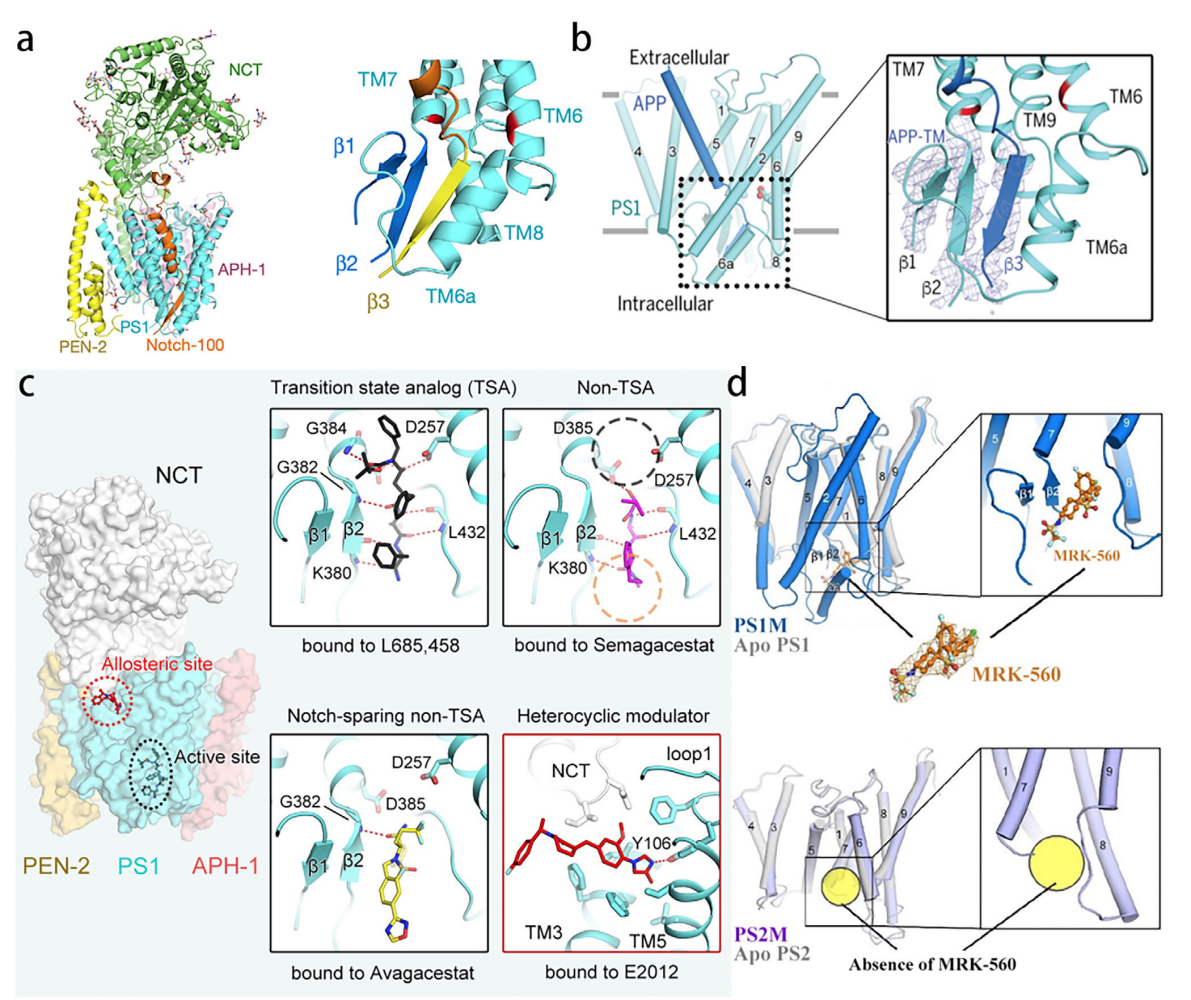# Structural Mechanism of γ‐secretase Cleavage and Regulation

**DOI:** 10.1002/alz.090513

**Published:** 2025-01-09

**Authors:** Rui Zhou

**Affiliations:** ^1^ Tsinghua university, Beijing, na China

## Abstract

Alzheimer’s Disease (AD) is characterized by the amyloid plaques in patient brain. The plaques are formed by β‐amyloid peptides (Aβs) that derive from the cleavage by γ‐secretase. Over 300 AD pathogenic mutations have been identified in presenilin1/2 (PS1/PS2), the catalytic subunit of γ‐secretase. Therefore, γ‐secretase serves as a potential drug target of AD. But inhibition of γ‐secretase suffers from severe side effect in clinical trials, which is thought to derive from unspecific inhibition towards its other substrates such as Notch. So, what is the molecular basis of γ‐secretase cleavage and how does the activity of γ‐secretase is regulated, especially in a substrate‐selective way?

To unravel the mechanism of substrate cleavage, we determined the cryo‐EM structures of γ‐secretase in complex with APP/Notch fragment, respectively. These structures reveal contrasting features of different substrates binding, thus providing valuable information for modulation of γ‐secretase cleavage in a substrate‐selective way. In addition, we solved structures of γ‐secretase bound to a number of inhibitors and modulator. Structural analysis reveals a set of shared themes and variations for inhibitor and modulator recognition that will guide development of the next‐generation substrate‐selective inhibitors. All these works lay the foundation for a better understanding towards the working mechanism of γ‐secretase and may facilitate related therapeutic intervention.